# P-269. Automated Interpretation of Hospital-Acquired Infection Rates: A Simultaneous Approach Using Both Monthly and Annualized Data

**DOI:** 10.1093/ofid/ofae631.473

**Published:** 2025-01-29

**Authors:** Bráulio R G M Couto, Ana Paula Ladeira, Walisson Ferreira Carvalho, Naísses Zóia Lima, Marcelo Esteves, Felipe De Paula, Bernardo Afonso, Rossana Souza

**Affiliations:** AMECI – Associação Mineira de Epidemiologia e Controle de Infecções, Belo Horizonte, Minas Gerais, Brazil; Biobyte Tecnologia em Epidemiologia, Belo Horizonte, Minas Gerais, Brazil; PUC MInas, Belo Horizonte, Minas Gerais, Brazil; PUC MInas, Belo Horizonte, Minas Gerais, Brazil; Biobyte EpiTech, Belo Horizonte, Minas Gerais, Brazil; Biobyte EpiTech, Belo Horizonte, Minas Gerais, Brazil; Biobyte EpiTech, Belo Horizonte, Minas Gerais, Brazil; Biobyte Sistemas, Belo Horizonte, Minas Gerais, Brazil

## Abstract

**Background:**

As more indicators are monitored by the infection control service, automating the interpretation of results becomes increasingly important; here, we propose a method that uses both short-term (monthly) and long-term (annualized) data to provide a comprehensive understanding of infection trends, including the detection of outbreaks.

**Figure 1:**
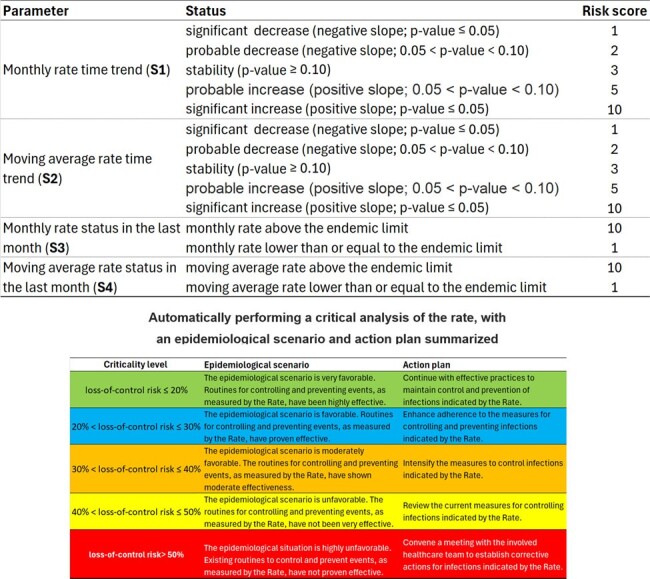
Criticality matrix for the indicator and automatic critical analysis of the rate. Criticality matrix for the indicator and automatic critical analysis of the rate.

**Methods:**

The automated critical analysis of a rate is conducted by simultaneously evaluating both the monthly rate and its moving average. While the month-by-month rate shows the indicator's current monthly results, the moving average indicates the overall trend, smoothing out noise from occasional aberrant values. A criticality matrix for the indicator is constructed:

Trend Analysis: Examine both the monthly rate and the moving average for trends. A p-value of up to 0.05 indicates a significant increase or decrease, while a p-value above 0.05 and below 0.10 suggests a probable increase or decrease. A p-value of 0.10 or higher indicates stability.

Last Month's Status: Compare the indicator's monthly value and moving average to their respective targets (90th percentile).

Generate risk scores for rate loss of control by combining the results of four analyses, yielding a total score *S*=*S*1+*S*2+*S*3+*S*4, and risk of loss of control is (*S*−4)/36×100%. This approach allows for automated analysis and interpretation of the epidemiological scenario (as illustrated in Fig. 1).

**Figure 2:**
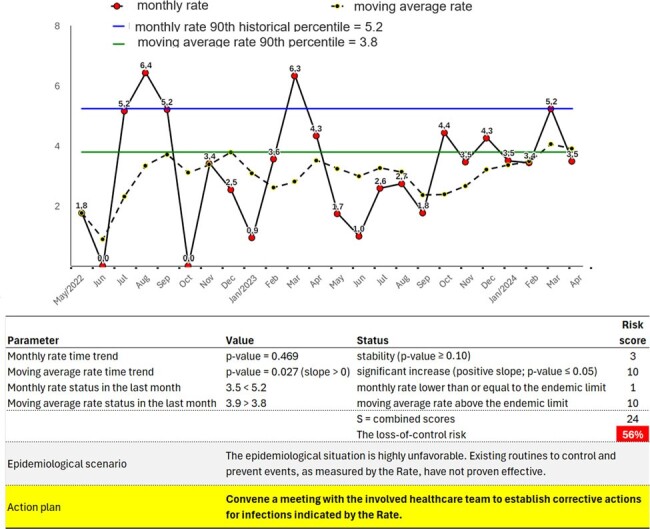
Automated critical analysis of the rate of hospital-acquired infections (HAI) per 1,000 patient-days caused by Acinetobacter baumannii resistant to carbapenems.

Automated critical analysis of the rate of hospital-acquired infections (HAI) per 1,000 patient-days caused by Acinetobacter baumannii resistant to carbapenems.

**Results:**

Figure 2 shows the rate of hospital-acquired infections (HAI) per 1,000 patient-days caused by Acinetobacter baumannii resistant to carbapenems. The automated analysis indicates a 56% risk of loss of control. Figure 3 illustrates the rate of central line-associated bloodstream infections (CLABSI) per 1,000 central-line days in an intensive care unit. The analysis indicates a 3% risk of loss of control. Figure 4 demonstrates the automated analysis of ventilator-associated pneumonia (VAP), with the number of VAP incidents per 1,000 ventilator-days, indicating an 11% risk of loss of control.

**Figure 3:**
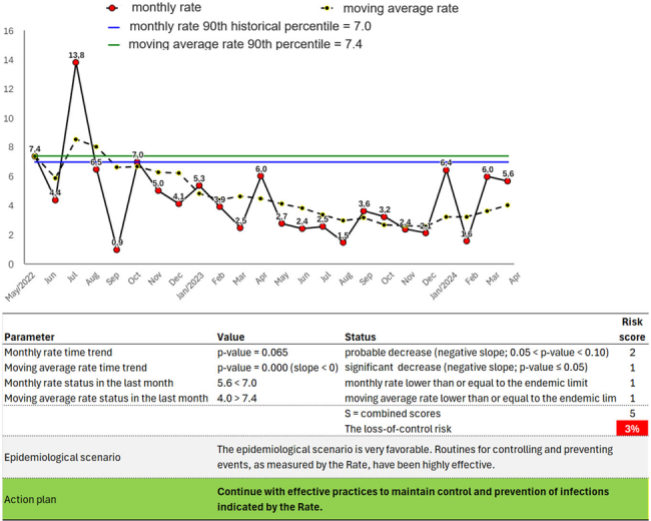
Automated critical analysis of the central line bloodstream infection (CLABSI) rate (#CLABSI/1,000 central-line days) in an intensive care unit.

Automated critical analysis of the central line bloodstream infection (CLABSI) rate (#CLABSI/1,000 central-line days) in an intensive care unit.

**Conclusion:**

The method allows automated critical analysis to effectively evaluate infection rates in healthcare settings. By combining short-term and long-term data, including monthly rates and moving averages, it offers a comprehensive view of infection trends.

**Figure 4:**
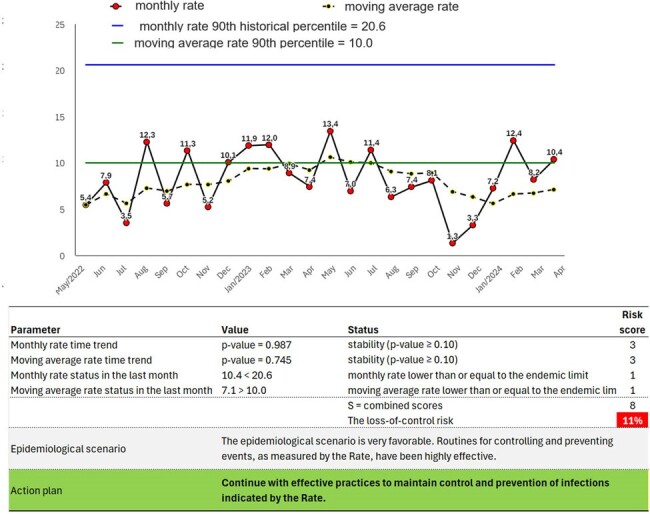
Automated critical analysis of ventilator-associated pneumonia (number of VAP incidents per 1,000 ventilator-days) in an intensive care unit.

Automated critical analysis of ventilator-associated pneumonia (number of VAP incidents per 1,000 ventilator-days) in an intensive care unit.

**Disclosures:**

**All Authors**: No reported disclosures

